# Can rare earth elements be recovered from abandoned mine tailings by means of electrokinetic-assisted phytoextraction?

**DOI:** 10.1007/s11356-024-32759-3

**Published:** 2024-03-08

**Authors:** Hassay Lizeth Medina-Díaz, Francisco Javier López-Bellido, Jacinto Alonso-Azcárate, Francisco Jesús Fernández-Morales, Luis Rodríguez

**Affiliations:** 1 Institute of Environmental and Chemical Technology (ITQUIMA), University of Castilla-La Mancha, Avenida Camilo José Cela, S/N, 13071 Ciudad Real, Spain; 2 School of Agricultural Engineering, University of Castilla-La Mancha, Ronda de Calatrava, S/N, 13003 Ciudad Real, Spain; 3 Faculty of Environmental Sciences and Biochemistry, University of Castilla-La Mancha, Avenida Carlos III, S/N, 45071 Toledo, Spain

**Keywords:** Rare earth elements, Phytoremediation, Electrokinetic, Mine tailings, Phytomining, Metal recovery

## Abstract

Given the high impact of traditional mining, the recovery of rare earth elements (REEs) from hazardous waste materials could become an option for the future in accordance with the principles of the circular economy. In this work, the technical feasibility of REEs recovery from metal mine tailings has been explored using electrokinetic-assisted phytoremediation with ryegrass (*Lolium perenne* L.). Phytoextraction combined with both AC current and DC current with reversal polarity was applied (1 V cm^−1^, 8 h day^−1^) to real mine tailings containing a total concentration of REEs (Sc, Y, La, Ce, Pr, and Nd) of around 146 mg kg^−1^. Changes in REEs geochemical fractionation and their concentrations in the soil pore water showed the mobilization of REEs caused by plants and electric current; REE availability was increased to a higher extent for combined electrokinetic-assisted phytoextraction treatments showing the relevant role of plants in the process. Our results demonstrated the initial hypothesis that it is feasible to recover REEs from real metal mining waste by phytoextraction and that the performance of this technology can be significantly improved by applying electric current, especially of the AC type, which increased REE accumulation in ryegrass in the range 57–68% as compared to that of the treatment without electric field application.

**Supplementary Information:**

The online version contains supplementary material available at 10.1007/s11356-024-32759-3.

## Introduction

Rare earth elements (REEs) have had an important role in the energy transition and technological development worldwide (Binnemans et al. 2013). REEs are considered excellent electrical conductors, making them key components of various products related to the high-tech industry such as low-carbon energy technologies, electric and hybrid vehicles, rechargeable batteries, wind turbines, and electronic devices (Ananth et al. 2009). Even, REE-enriched fertilizers have recently been implemented in the agricultural field to improve crop growth and agronomic performance (Thomas et al. 2014; Ramos et al. 2016).

This group of essential metals is mainly comprised of 15 lanthanides that are divided into two groups: light REEs (LREE: La, Ce, Pr, Nd, Pm, Sm, and Eu) and heavy REEs (HREE: Gd, Tb, Dy, Ho, Er, Tm, Yb and Lu), plus Y and Sc (Liu et al. 2021). REEs are widespread in the Earth crust and their concentration may even be higher than those of other common metals (Dinh et al. 2022); they are usually found in the chemical form of oxides, phosphates, carbonates and silicates (Tao et al. 2022). The most feasible REEs minerals for extraction are bastnaesites, monazites and xenotimes, which contain up to 65–75% REEs (Wübbeke 2013). Unfortunately, the negative environmental impacts associated with conventional extensive mining methods include the use of chemical compounds, expensive equipment required and the release of wastes (Massari and Ruberti 2013; Dinh et al. 2022). The extraction and separation of the different REEs from their sources are a complex and difficult process because of their similar physical and chemical properties (García et al. 2020; Wen et al. 2021). In most cases, REE extraction is more profitable as a by-product of mining other metals (Wübbeke 2013), since they are usually not found in sufficient abundance in a single location to make their exploitation economically cost-effective (Krzciuk and Gałuszka 2015). Sizeable REE deposits are mainly distributed in China, Russia, Brazil, Australia, and Vietnam (Charalampides et al. 2015; Lima and Ottosen 2021). REEs have been included in the European Commission’s list of critical raw materials as a consequence of their economic relevance and the depletion of resources (Martins and Castro 2020). In this sense, the pursuit of potential sources of these strategic elements in Europe has become a relevant issue (Balaram 2019). Hence, waste recycling is presented as a viable alternative in terms of sustainability and circular economy, which has been poorly implemented (Almeida et al. 2021; Dang and Li 2022); in fact, currently, barely 2.8% of the total REEs waste discarded is recycled (Lima and Ottosen 2021). Consequently, mining wastes from metallic ores could be a valuable source for the recovery of REEs and a suitable initiative for treating this type of stockpiled and abandoned wastes at mining sites (Goodenough et al. 2016; Van der Ent et al. 2021; Hernández et al. 2023).

In this frame, phytoremediation technologies emerges as a cost-effective, environmentally sustainable and promising alternative for the rehabilitation of abandoned mining sites which would allow, not only the removal of metals from polluted soil and waste, but also the recovery of valuable elements from low-grade ores or metal-rich soils (Ashraf et al. 2019; Khorasanipour and Rashidi 2020). Phytoremediation involving metal recovery is usually called as “phytomining.” Although REEs are not essential elements for plants, they can be accumulated into their tissues at moderated concentrations (Grosjean et al. 2019); to date, phytomining studies have mainly been focused on the extraction of major metals (Cu, Zn, Cd, and Pb) (Salas-Luévano et al. 2017; Zhang et al. 2019; Gascó et al. 2019), but they have scarcely been applied for REEs recovery (Jalali and Lebeau 2021). One of the most cited drawbacks of phytoextraction is the relative slowness of the technique, mainly due to the low availability of metals in the solid matrix and, consequently, the reduced ability of plants for metal uptake. Among the different approaches that have been studied in order to increase phytoextraction efficiency, one of the most interesting and novel is the combination of phytoextraction and electrokinetics, named as electrokinetic-assisted phytoremediation (EKPh). This technique has been proven to significantly increase phytoextraction yields for the uptake of major metals from soils and waste (Zhou et al. 2007; Acosta-Santoyo et al. 2017), but, to our best knowledge, its application for the recovery of REEs has not yet been studied.

In this context, the present research shows the results obtained in the application of electrokinetic-assisted phytoextraction to recover rare earth elements from real multi-metal polluted mine tailings. EKPh experiments were carried out using a growth substrate constituted of tailings from a former Pb/Zn mine, the plant ryegrass (*Lolium perenne* L.), and two types of electric field application, i.e., alternate current and direct current with reversal polarity. Performance of the experiments has been followed by analyzing different physicochemical parameters of the liquid and solid phases of the growth substrate along with the REE concentration in the plant tissues. Ryegrass has been selected because of its capability to grow quickly and developing a considerable biomass in this type of polluted substrate along with its recognized ability to accumulate metals in their tissues (Houben et al. 2013). The use of real waste from an abandoned mine site adds novelty to this work and may allow a first assessment of the feasibility of applying this technology for the recovery of resources from this type of waste.

## Materials and methods

### Studied area and sampling

The research utilized mine waste materials obtained from the San Quintin Mine, an inactive mining site in the South-Central region of Spain. The specific location of the mine site can be identified using UTM coordinates: 389484, 4297643. This particular mine was once actively exploited to extract galena (PbS) and sphalerite (ZnS) from the late nineteenth century until the first half of the twentieth century. Detailed information about the mine site can be found in previous studies conducted by Higueras et al. (2012) and Rodríguez et al. (2009). The sample of mine tailings was collected from a depth of 60 cm and subsequently air-dried under ambient conditions at the laboratory. To ensure homogeneity, the sample was sieved to a particle size of 2 mm and blended with washed sand in a 75:25 weight ratio. This prepared mixture served as the growth substrate for the EKPh experiments. Essential information regarding the concentration of rare earth elements, major metals, and various physicochemical characteristics of the growth substrate can be found in Table 1.

**Table 1:** Physicochemical properties and total concentrations of REEs and major metals in the growth substrate (mine tailings/sand 75:25 w:w) used in the experiments

Parameter	Value	Analytical method
pH	6.80 ± 0.05	Soil–water ratio of 1:5 (w:v)
Electrical conductivity (mS cm−1)	0.66 ± 0.02	
% Inorganic carbon	0.01	Shimadzu TOC Multi EA 4000 carbon analyzer
% Organic carbon	0.08	
% Total carbon	0.09	
% Sand	30.4 ± 1.6	Bouyoucos-hydrometer method
% Clay	1.7 ± 0.9	
% Silt	67.9 ± 1.6	
Texture (USDA classification)	Silt loam	
Total Pb, mg kg−1	3832 ± 122	Acid digestion and inductively coupled plasma mass spectrophotometer (ICP-MS)
Total Zn, mg kg−1	2904 ± 132	
Total Cu, mg kg−1	93.99 ± 3.7	
Total Cd, mg kg−1	38.68 ± 4.0	
Total Ce, mg kg−1	59.54 ± 3.33	
Total La, mg kg−1	32.48 ± 2.52	
Total Nd, mg kg−1	24.76 ± 1.44	
Total Pr, mg kg−1	7.390 ± 0.41	
Total Cs, mg kg−1	6.174 ± 0.63	
Total Y, mg kg−1	15.34 ± 0.92	

### Experimental design and methodology

**Fig. 1:**
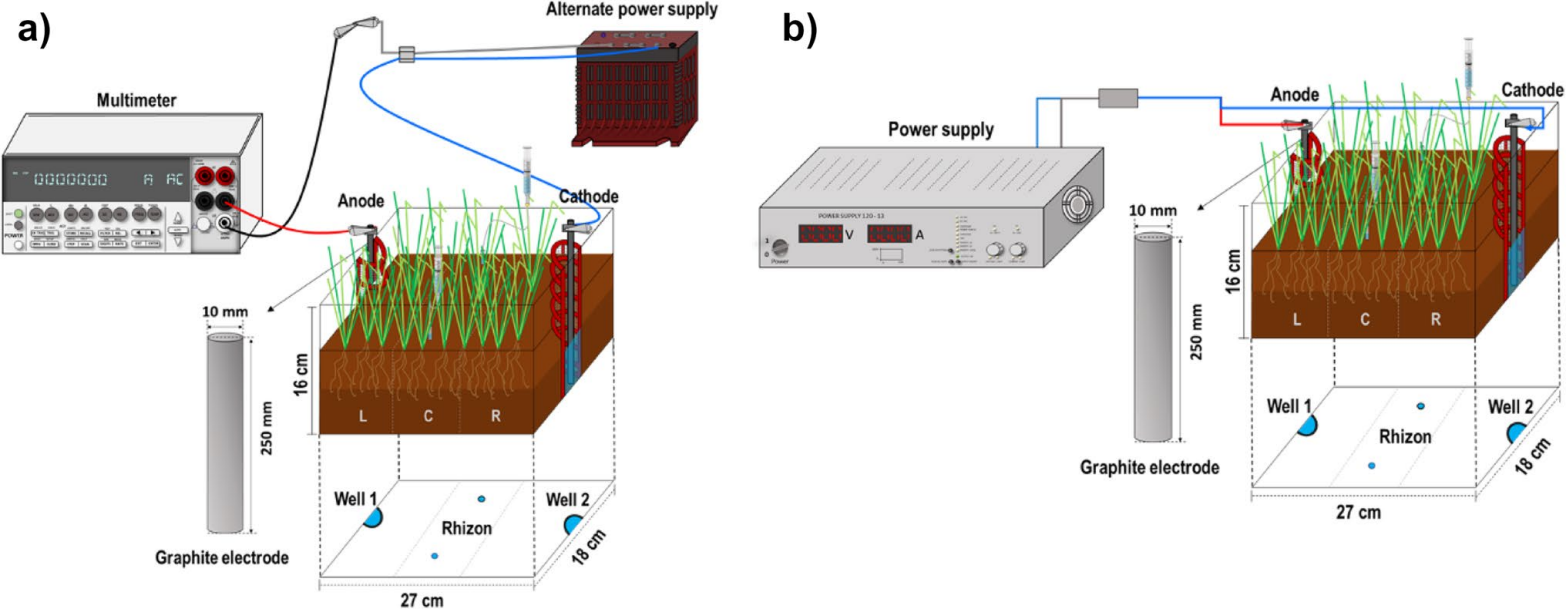
Schematic representation of the experimental setup used in the electrokinetic-assisted phytoextraction tests: **a** alternate current treatments; **b** direct current treatments

For the EKPh experiments, rectangular plastic containers made of transparent material were used. These containers measured 27 cm in length, 17 cm in width, and 18 cm in height. Each container had semi-cylindrical wells with nylon mesh walls (120 mesh, 5 cm in diameter) located at both ends. To set up the electrodes, graphite rods measuring 1 cm in diameter and 25 cm in height were placed inside these wells (Fig. 1).

Seeds of English or perennial ryegrass (*L. perenne*) were then sown into the containers using a dosage of 100 g m^−2^. To be precise, each container contained 7 kg of growth substrate. The containers were subsequently fertilized with a solution containing nitrogen, phosphorus and potassium using a rate of 40 kg N ha^−1^, 30 kg P_2_O_5_ ha^−1^, and 90 kg K_2_O ha^−1^.Additionally, they were irrigated daily with tap water to maintain moisture at field capacity, representing 28.5% of the dry weight of the substrate. The water level in the electrode wells was monitored and adjusted as needed. Initially, the water level measured approximately 8 cm.

The plants were allowed to grow for 44 days without the application of electric current. Following this period, an electric current was applied for an additional 14 days. The EKPh experiments were conducted in a greenhouse equipped with artificial lighting, providing 12 h of light per day (from 8:00 a.m. to 6:00 p.m.). The greenhouse also maintained controlled temperatures, ranging between 22 and 29 °C during the day and 11–14 °C at night.

Five different treatment groups were implemented for the experiments: (a) ryegrass under alternating current (EKPhAC series); (b) ryegrass under direct current with polarity reversal (EKPhDC series); (c) unseeded substrate with alternating current (EKAC series); (d) unseeded substrate with direct current and polarity reversal (EKDC series); and (e) ryegrass without the application of electric current (PHYTO series). The experimental design followed a randomized block layout, with three replicates for each treatment.

A constant voltage gradient of 1 V cm^−1^ was applied between days 45 and 58, for 8 h per day (from 10:00 a.m. to 6:00 p.m.). That dose of electric current was selected based in previous papers with the objective of increasing the mobility of REEs without affecting plant growth (Rodríguez et al. 2022). This was achieved using a DC power supply (Delta Elektronica S.V., model SM120-13, The Netherlands) for the EKDC and EKPhDC series, while an AC power supply (Polylux, model TRAFO QB200, Spain) was used for the EKAC and EKPhAC series. The electrode that initially operated as the anode was labelled as W1, while the other electrode was labelled as W2. In the experimental series involving DC current, the polarity of the electric field was switched 4 h after the start of the application (2:00 p.m.), resulting in the reversal of electrode roles.

### Sample collection and analysis

To determine the concentration of dissolved REEs in the liquid soil matrix and electrode well solutions, periodic extraction and subsequent analysis were performed. A total of five samplings were conducted on days 46, 49, 52, 55, and 58. The liquid soil matrix was sampled using Rhizon samplers, while the electrode well solution was also analyzed.

Samples of the growth substrate located in the central section of the containers were taken at the end of the EKPh experiments. These solid samples were air-dried at room temperature for 24 h before being ground in a ball mill (Retsch model MM200, Germany) for further analysis. Plant samples were also collected after the EKPh experiments and separated into shoots and roots. The plant samples were washed with deionized water and then dried in an oven at a temperature of 80 °C for 24 h. Finally, they were ground using an ultra-centrifugal mill (Retsch model ZM 200, Germany).

All the solid samples, including the growth substrate and plant samples, were digested using the EPA3051A method. This involved mixing 0.5 g of ground sample with 9 mL of concentrated nitric acid and 3 mL of concentrated hydrochloric acid. The mixture was then digested in a microwave digestor. For the determination of the geochemical distribution of REEs in the mine tailings, the modified BCR sequential extraction method was utilized. This method, described by Rodríguez et al. (2009), allowed for the extraction of four metal fractions: exchangeable and acid-soluble, bound to Fe/Mn oxides, bound to organic matter and sulfides, and the residual fraction. Details of the extraction method can be found in Table SM1 in the Supplementary Materials.

The concentrations of REEs, specifically La, Ce, Pr, Nd, Y, and Sc, in the liquid samples (including soil solution samples and the extracts from digestion and BCR extraction) were analyzed using an ICP-MS spectrophotometer (Thermo iCAP TQ model from ThermoFisher Scientific, Waltham, MA). To ensure the quality assurance of the REE analysis, comparisons were made with standard reference materials such as SQC001 (Sigma-Aldrich) for soil samples and ERM-CD281 (European Reference Materials) for plant samples. The accuracy of the BCR sequential extraction method was assessed using the BCR-701 standard reference material. Differences up to 10% between measured values and certified ones were obtained. Additionally, blank control samples were included in the analysis, with acid digestions being checked every 10 samples, and the sequential extraction procedure being checked every 9 samples.

### Statistical analysis

For analyzing the data, the Statistix 10 package (Analytical Software, USA) was employed. The analysis of variance (ANOVA) was conducted on each parameter measured, following the experimental design of randomized complete block with three replicates. In order to compare the average values of these parameters, Fisher’s least significant difference (LSD) test was utilized, at a significance level of *p* ≤ 0.05 as specified by Gómez and Gómez (1984). The statistical differences between the mean values were then depicted in all tables and figures by assigning different letters to indicate these distinctions.

## Results and discussion

### Changes in the REE geochemical fractionation in mine tailings

The most abundant REEs detected in the mine tailings used in this research corresponded to Ce, La, and Nd, with mean concentrations of 59.54, 32.48, and 24.76 mg·kg^−1^ respectively (Table 1); the order in REE concentrations was Ce > La > Nd > Y > Pr > Sc. The distribution pattern found for the LREEs (La, Ce, Pr, and Nd) aligns with the Oddo-Harkins rule, which establishes that the elements with even atomic numbers tend to exhibit higher abundance compared to their neighbors with odd atomic numbers (Loell et al. 2011). Overall, the total concentration of REEs found in the studied mine tailings, around 146 mg·kg^−1^, was significantly lower than those reported for contaminated soils from nearby locations of REEs mine tailings in China, which ranged from 156 to 56,500 mg·kg^−1^; nevertheless, it closely approximates the mean concentration found in the Chinese soils (181 mg·kg^−1^), which serves as a primary reference for the lanthanide reserves in the world (Wang and Liang 2015).

**Fig. 2:**
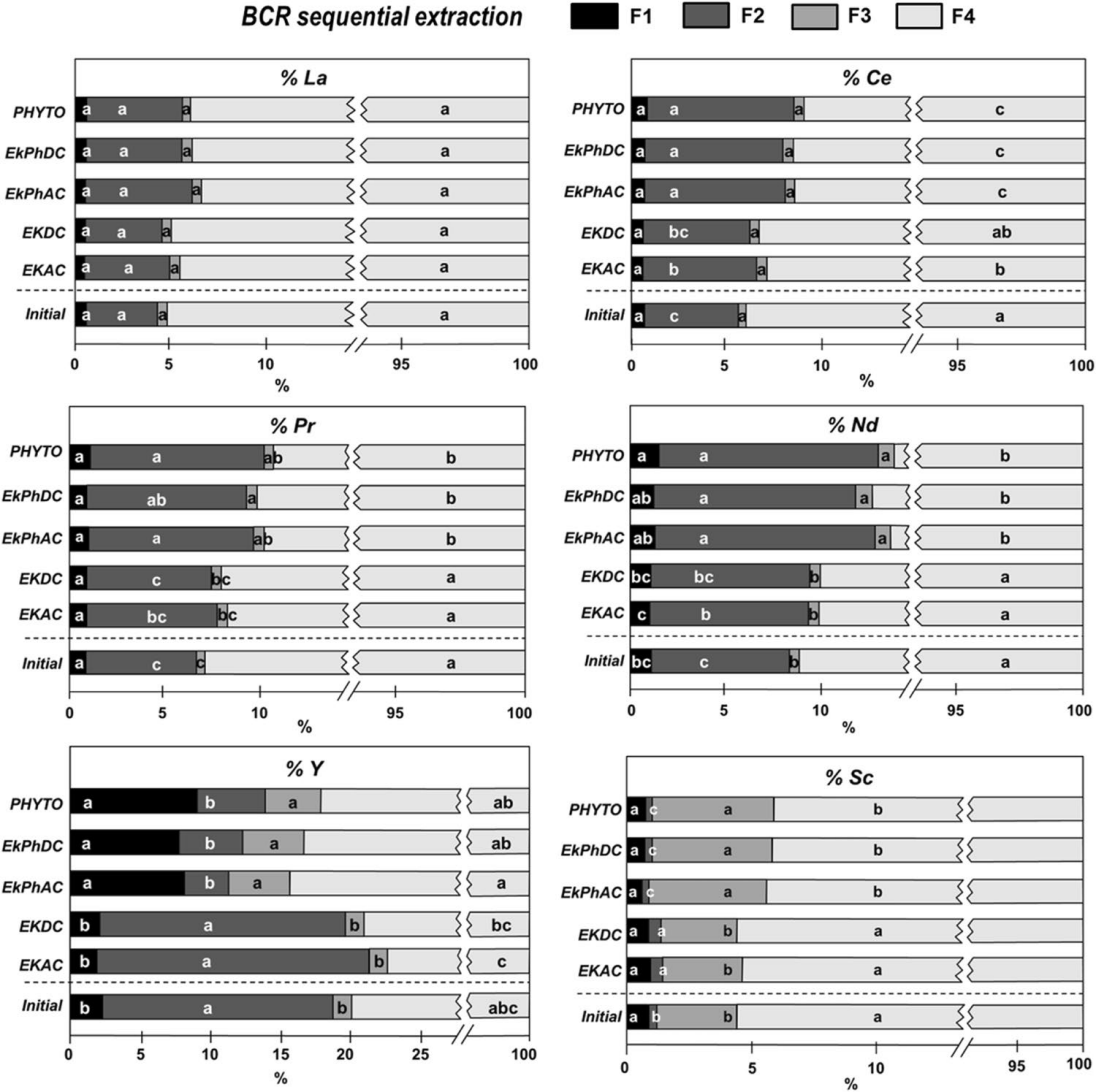
Geochemical fractionation of REEs (expressed as % of the total concentration) in the initial mine tailings substrate (mine tailings/sand 75:25 w:w) and after the application of the different treatments obtained by the BCR sequential extraction method: (F1) exchangeable and acid-soluble REEs, (F2) REEs bound to Fe/Mn oxides, (F3) REEs bound to organic matter and sulfides and, (F4) REEs in residual fraction. Treatments applied: PHYTO: plants and no electricity; EKDC and EKAC: electric current and no plants; EKPhDC and EKPhAC: plants and electric current. Distinct letters indicate significant differences between treatments (*n* = 3; Fisher’s LSD test; *p* ≤ 0.05) for each REE and fraction (F1, F2, F3, and F4)

The geochemical fractionation of REEs (determined using the BCR sequential extraction method) in the initial growth substrate and in the samples taken at the end of the EKPh experiments was obtained to assess the geochemical changes occurred in the mine tailings after the application of the different treatments; the results are shown in Fig. 2. The initial geochemical distribution of REEs was predominantly dominated by the fraction bound to the residual fraction (F4), comprising approximately 92% of the total REEs, and to a lesser extent, by the fraction bound to Fe/Mn oxides (F2), accounting for approximately 6.2%. A similar affinity of REEs was reported by Liu et al. (2016) for the geochemical fractionation in sediments, showing that REEs could be bounded to Fe/Mn oxides although Fe oxide minerals were relatively low; similar findings were also found for soils (Jalali and Lebeau 2021). Other studies showed that the residual fraction generally represents up to 90% of the REE concentration, and it is typically composed of aluminosilicates (small-sized ores that mainly constitute clays) and phosphates (Lima and Ottosen 2021); moreover, this fraction is considered the least mobile and available fraction for plant uptake (Malsiu et al. 2020). Likewise, the mobility and solubility of REEs are strongly influenced redox potential and pH (Laveuf et al. 2012). In our case, soil pH was kept around neutral values even when the electric current was applied and the electrochemical redox reactions took place in the electrodes (Medina-Díaz et al. 2023); hence, under these conditions the mobility of REEs may be fairly restricted (Wiche et al. 2017). Likewise, the adsorption capacity of cations is significantly influenced by their valence and hydrated ionic radius (Cuevas Durán 2010; Khorasanipour and Rashidi 2020); as the size decreases and the valence increases, REE and other metal ions are held more strongly (Kabata-Pendias 2010). This trend explains the order found for REEs F1 and F2 fractions in the used mine tailings, i.e., Y > Nd > Pr > Ce > La > Sc; with the exception of Sc (which showed a high affinity for organic matter, as will be seen later), that order is just the opposite to that of atomic size.

Some significant changes were observed in the different REEs fractions of the mine tailings after the application of electric current and/or plants (Fig. 2). The residual fractions (F4) of Ce, Pr, Nd, and Sc were significantly decreased, as compared to the initial values, by the application of phytoremediation and EKPh treatments; mean percentages also decreased for the residual fraction of La, but that decrease was not significant. However, the Y residual fraction increased under these same treatments; since electrokinetic treatments without plants did show the decrease of the residual fraction, this rise may be associated with the uptake of more labile fractions by plants for this element (see the “Ryegrass biomass and phytoextraction of REEs” section). The electrokinetic treatments without plants were less effective for the REE mobilization; significant decreases in the residual fraction were only found for Ce and Y under AC current application (EKAC series). It means, on one hand, that the application of treatments based on phytoremediation achieved to increase the availability of metals in the mine tailings; on the other hand, these results also reveal the synergistic effect of the combination of electrokinetics and phytoremediation. The good performance on metal mobilization by the PHYTO treatment can be attributed to the secretion of plant metabolites (root exudates), such as organic acids, as it was previously suggested by us (Medina-Díaz et al. 2023).

Ce, Nd, and Pr concentrations of the F2 fraction were significantly increased, with respect to the initial value, for the PHYTO, EKPhDC, and EKPhAC treatments; on the contrary, the application of electrokinetics without plants only increased in a significant way the F2 fraction of Sc (Fig. 2). Likewise, Sc and Y F2 concentrations were significantly decreased under the effect of the single and combined phytoextraction treatments. It has been previously reported that the Fe/Mn oxides have a relevant role in the release and availability of REE compounds (Thomas et al. 2014); this is a process mainly favored under reducing conditions which strongly depends on redox potential and pH (Laveuf et al. 2012; Liu et al. 2016). Ce is the REE most sensitive to redox potential changes, since Ce(III) can be easily oxidized to Ce(IV) reducing its availability for plants (Wang and Liang 2015). On the other side, REEs were poorly bounded to oxidizable BCR fraction (F3), except for Sc (Fig. 2). Even though, other studies have indicated that REEs may further exhibit a strong affinity for organic matter (Wiche et al. 2017; Vukojević et al. 2019), in this study, the complexation between REEs and organic matter could be restricted due to the low organic carbon content (< 1%) of mine tailings (Liu et al. 2020), which is a typical characteristic of this type of waste (Tao et al. 2022). Nevertheless, Pr, Nd, Y, and Sc F3 concentrations were significantly increased with respect to the initial mine tailings under EKPhAC, EKPhDC, and PHYTO treatments (Fig. 2). Lastly, the F1 fraction showed low values for most of the studied REEs; it consists as weak-adsorbed free ions on the soil particles surface, susceptible to ion exchange, along with metals bound to carbonates; this is considered the most soluble and mobile fraction (Kotelnikova et al. 2020). Significant changes were found for Y and Nd, whose F1 fraction increased significantly under the application of combined and phytoextraction treatments, respectively (Fig. 2).

### Distribution of REE concentration in water in soil pores and electrode wells

**Fig. 3:**
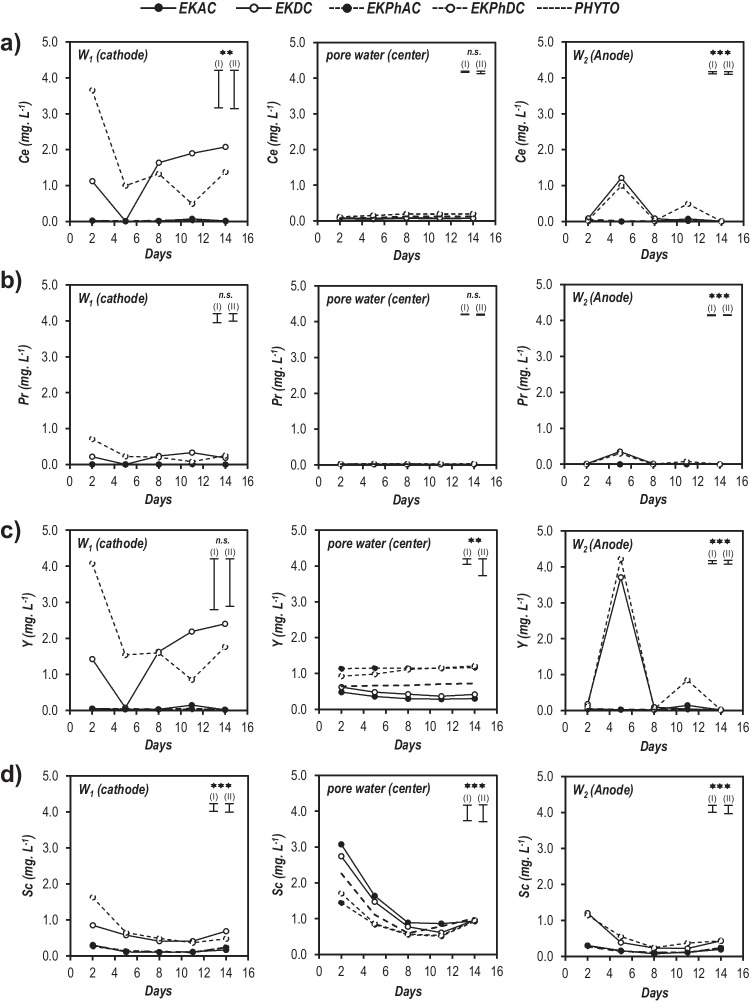
REEs concentrations (mg L^−1^) in the water obtained from electrode wells (W_1_ and W_2_) and the soil pores (collected from the middle section of each container by Rhizon samplers) sampled (*n* = 3) just before turning off the electric current on different days: **a** Ce; **b** Pr; **c** Y; **d** Sc. Treatments applied: PHYTO: plants and no electricity; EKDC and EKAC: electric current and no plants; EKPhDC and EKPhAC: plants and electric current. For each REE and section; single, double, and triple asterisks indicate significant level (two-way ANOVA analysis) of “concentration × time” interaction. Vertical bars show LSD test (*p* ≤ 0.05): (I) for same level of EK/Phyto treatment and (II) for different levels of treatments

The behavior REEs in the solution of the soil pores and electrodes (W_1_ and W_2_) was assessed in order to detect changes in their solubility and mobility; concentrations of Ce, Pr, Y, and Sc over the days are shown in Fig. 3 (La and Nd concentrations were also measured and, since their similarity with Ce, they have been included in the Figure SM1 of the Supplementary Materials). With respect to the electrode wells, the highest concentrations of REEs were detected for the treatments using DC current; it was expected because electroosmotic and electromigration fluxes can only be established using this type of electric field (Medina-Díaz et al. 2023). The anode–cathode movement induced by electroosmosis along with the cationic form of REEs, which allowed their transport to the opposite-charge electrode well (cathode, W1) (Sánchez et al. 2018; Cameselle et al. 2019), may have contributed to obtain the highest concentrations of REEs in the cathode well under treatments using DC current (Luo et al. 2018; Awa and Hadibarata 2020). The presence of significant amounts of Ce, Y, and Sc in the anode (W2) can be explained by the partial dissolution of some metal species in the vicinity of the anode due to the low pH caused by water electrolysis (Medina-Díaz et al. 2023). Between metals, Ce and Y reached the highest concentrations in both in the anode and cathode; it can be attributed, on one hand, to the high availability of Y in the mine tailings (in agreement with that seen in the previous section) and, on the other hand, to the higher content of Ce (as compared to Pr, Sc, and Y) in the used mine tailings. Likewise, it was observed that the REE concentrations decreased progressively under the EKPhDC treatment but was raised under the EKDC treatment; it means that the presence of plants affected the concentration of soluble REEs and their migration towards the electrode wells.

Regarding the soil pore water extracted by the Rhizon samplers, concentrations of Ce and Pr (mean values of 0.116 and 0.021 µg L^−1^, respectively) were approximately three times lower than those of the electrode wells (0.337 and 0.059 µg L^−1^ for Ce and Pr, respectively) in all treatments (Fig. 3). On the contrary, Y and Sc concentrations were quite higher than Ce and Pr ones for all treatments (mean values of 0.741 and 1.149 µg L^−1^ for Y and Sc, respectively); moreover, Y concentration remained almost constant while Sc concentration decreased as the experiment progressed. Both in electrode wells and soil pore water, Y and Sc highlighted heterogeneous behavior with respect to other REEs, possibly due to their lower ionic radius (Ou et al. 2022; Galhardi et al. 2022). The high solubility of Y is in agreement with that seen for its geochemical partitioning in the previous section; solubility of Sc can be attributed to the formation of the Sc(H_2_O)_6_^3+^ and Sc(H_2_O)_5_OH^2+^ complexes in aqueous phases along with its affinity for complexing with sulphates and carbonates (Kabata-Pendias 2010; Liu et al. 2016; Kotelnikova et al. 2021). As pointed out in the previous section, REEs are usually predominantly adsorbed to the solid phase of the soil and just a tiny amount is dissolved in the soil interstitial water (Tao et al. 2022). In general, the low soluble REE concentrations obtained (< 1 mg L^−1^), in consequence of the low availability of REEs in the mine tailings used here (the “Changes in the REE geochemical fractionation in mine tailings” section), are consistent with other research (Kotelnikova et al. 2020) and those values found in natural waters (Migaszewski and Gałuszka 2015). In general, the results obtained from the REE concentrations in the liquid samples showed that the substantially low availability of the REEs can be improved by applying electric current, especially DC current. (Liu et al. 2016; Yuan et al. 2021). Furthermore, although REEs are not considered essential elements for plants, the soluble forms can be absorbed through the root system, being strongly retained in the negatively charged cell wall (Khan et al. 2017). In fact, as will be seen in the next section, Sc accumulated poorly in the ryegrass tissues; this could be the reason why significant concentrations of this element remained in the soil solution throughout the experiments.

### Ryegrass biomass and phytoextraction of REEs

The biomass production of ryegrass was assessed after the end of the experiments (Figure SM2 in Supplementary Materials). Dry weight of ryegrass was not significantly different for the combined treatments (EKPhDC and EKPhAC series) as compared to the phytoextraction one (PHYTO); average biomass values of 4.04, 7.21, and 11.25 g per container were obtained for roots, shoots, and total plant, respectively. It suggests that the ryegrass growth was not affected when either DC or AC electric fields were applied. Our results contrast with some previous studies that reported enhanced effects on plant growth under AC current, due to the biological/biochemical alterations induced in response to the interaction between electric current and interstitial fluid (Cang et al. 2011; Cameselle et al. 2013; Acosta-Santoyo et al. 2017). Likewise, the improvement of enzymatic activities capable to reduce oxidative stress conditions (He et al. 2017; Kovaříková et al. 2019) and the mobilization of plant nutrients in the rhizosphere vicinity thanks to the electrokinetic process at moderate voltages have also been reported (Lima et al. 2017). On the other hand, other authors affirm that plant growth may be stimulated when low doses of REEs are accumulated into plant tissues, but its toxicity may increase along with their concentration (Jalali and Lebeau 2021). However, in our study, all these potential beneficial effects for ryegrass growth were masked by the high toxicity caused by the high concentrations of metals such as Pb or Cd existing in the mine tailings used as a growth substrate (Medina-Díaz et al. 2023); in fact, chlorosis and early wilting symptoms were observed for all treatments.

**Fig. 4:**
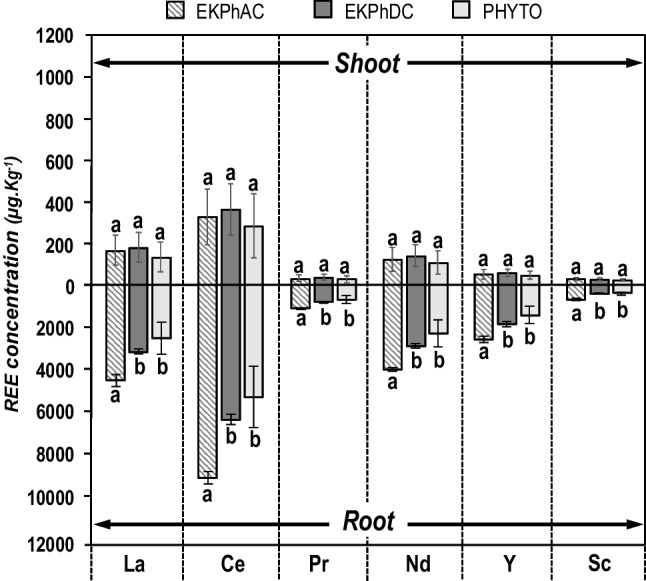
REEs concentration (µg kg^−1^) in ryegrass tissues (roots and shoots) for the different treatments (*n* = 3; mean values ± STD). Treatments applied: PHYTO: plants and no electricity; EKDC and EKAC: electric current and no plants; EKPhDC and EKPhAC: plants and electric current. Distinct letters indicate significant differences between treatments (Fisher’s LSD test; *p* ≤ 0.05). Standard deviation (STD) is represented by vertical bars

Total concentrations of La, Ce, Pr, Nd, Sc, and Y accumulated in plant tissues after the application of the different treatments along with the total plant uptake values are shown in Table 2. The accumulation order of REEs in the ryegrass tissues followed the sequence Ce > La > Nd > Y > Pr > Sc, which is with the exception of Y consistent with the order of abundance of the REEs in the studied mine tailings. However, ryegrass showed different affinities for the different rare earth elements as it can be pointed out by calculating the bioconcentration factor (BCF, calculated as the ratio of the element concentration in the ryegrass root by that of the substrate). Considering this parameter, the order of REE uptake was Y > Ce ≈ Nd ≈ Pr > La >  > Sc; this trend clearly agrees with that obtained for the sum of F1 and F2 BCR fractions in the mine tailings substrate (the “Changes in the REE geochemical fractionation in mine tailings” section). Therefore, it seems that REEs belonging to those geochemical fractions are the most significant for the ryegrass availability; since the F3 fraction was found to be the majority in the case of Sc (the “Changes in the REE geochemical fractionation in mine tailings” section), its low accumulation in ryegrass would indicate that this fraction is not as available as F1 and F2 ones. Regarding the accumulation of REEs in the different plant tissues, all the elements were mainly accumulated in the roots with mean concentrations exceedingly more than 23 times the shoot ones (note the remarkable difference in the scales of the Y axis for root and shoot REE concentrations in Fig. 4). The REE concentrations reached in total biomass by ryegrass in this study were higher than those detected by Hu et al. (2022), who reported concentrations of 400–1400 µg kg^−1^ and 800–2100 µg kg^−1^ for La and Ce, respectively, in alfalfa (*Medicago sativa* L.) and 400–600 µg·kg^−1^and 600–1300 µg·kg^−1^ for La and Ce, respectively, in ryegrass (*L. perenne*). In the same way, our findings indicate significantly higher concentrations of La, Ce, Pr, and Nd in comparison to the study conducted by Anawar et al. (2012), in *Vicia villosa* Roth (223, 299, 63, and 261 µg kg^−1^ for La, Ce, Pr, and Nd, respectively) and *Camelia sinensis* (L.) Kuntze (393, 369, 103, and 421 µg kg^−1^ for La, Ce, Pr, and Nd, respectively). The relatively low concentrations and BCF values obtained here (in the range 0.01–0.24; Table 2) can be attributed, on the one hand, to the very reduced availability of REEs in the studied mine tailings (the “Changes in the REE geochemical fractionation in mine tailings” section) and the high total concentrations of major metals such as Pb or Zn (Table 1) which compete for metal uptake (Kovaříková et al. 2019). On the other hand, light REEs such as Ce, La, and Nd also compete with Ca^2+^ (macronutrient) for a similar transport pathway in a range of plant biological processes (Thomas et al. 2014); this is mainly due to the comparable ionic radius and higher charge density and trivalent form of REEs relative to Ca^+2^ (Hoshino et al. 2016; Jalali and Lebeau 2021). Previous studies reported that up to 80% of the REEs were concentrated in the root system (Khan et al. 2017). This is partly related to the selective uptake of REEs into root cell walls in the form of trivalent cations, which is one of the main REEs fixation mechanisms in plants (Tao et al. 2022). Once inside the roots, the translocation of REEs to the aerial parts is promoted mainly through the xylem, since its cells have a high cation exchange capacity that stimulates the binding of REEs with the ligands present in the inner membrane (aspartic acid, glutamic acid, citric acid, malic acid, and histidine) (Jalali and Lebeau 2021)*.*

**Table 2:** REEs concentrations in total plant (µg kg^−1^), REEs total uptake (µg per container), and bioconcentration factors (BCF, calculated as the ratio of REEs root concentrations by their concentration in soil) under the influence of different treatments (PHYTO, EKPhDC, and EKPhAC). All values are expressed in mean values ± STD. Different letters denote significant differences between treatments (*n* = 3; Fischer’s LSD test; *p* ≤ 0.05)

Parameter	PHYTO	EKPhDC	EKPhAC
Total plant concentration (µg kg^−1^)			
La	1034 ± 255 **b**	1252 ± 81.7 **b**	1757 ± 63.6 **a**
Ce	2180 ± 504 **b**	2546 ± 180.3 **b**	3572 ± 75.1 **a**
Pr	267.6 ± 64.8 **b**	319.7 ± 24.6 **ab**	440.9 ± 8.6 **a**
Nd	934.2 ± 222 **b**	1133 ± 89.1 **ab**	1549 ± 28.3 **a**
Y	571.0 ± 147 **b**	711.7 ± 73.9 **ab**	984.5 ± 48.2 **a**
Sc	159.1 ± 28.1 **b**	167.2 ± 15.1 **b**	278.8 ± 17.5 **a**
Total REEs uptake (µg per container)			
La	11.98 ± 2.95 **b**	13.70 ± 1.24 **b**	19.43 ± 1.74 **a**
Ce	25.23 ± 5.50 **b**	27.84 ± 2.40 **b**	39.53 ± 3.94 **a**
Pr	3.09 ± 0.71 **b**	3.49 ± 0.30 **b**	4.88 ± 0.51 **a**
Nd	10.80 ± 2.40 **b**	12.39 ± 0.99 **b**	17.15 ± 1.77 **a**
Y	6.57 ± 1.49 **b**	7.76 ± 0.74 **b**	10.90 ± 1.29 **a**
Sc	1.84 ± 0.25 **b**	1.83 ± 0.26 **b**	3.09 ± 0.38 **a**
Bioconcentration factor			
La	0.09 ± 0.02 **b**	0.11 ± 0.01 **b**	0.17 ± 0.01 **a**
Ce	0.11 ± 0.03 **b**	0.13 ± 0.001 **b**	0.19 ± 0.01 **a**
Pr	0.11 ± 0.02 **b**	0.12 ± 0.008 **b**	0.18 ± 0.01 **a**
Nd	0.12 ± 0.02 **b**	0.13 ± 0.007 **b**	0.19 ± 0.01 **a**
Y	0.14 ± 0.01 **b**	0.17 ± 0.002 **b**	0.24 ± 0.004 **a**
Sc	0.010 ± 0.002 **b**	0.013 ± 0.002 **b**	0.026 ± 0.004 **a**

In general terms, it was observed that the application of electric current increased the average concentrations of all the REEs accumulated in ryegrass shoots, roots and total plant as compared to those obtained in the phytoextraction treatment (Table 2; Fig. 4). However, those increases were not statistically significant in all the cases. Specifically, shoot concentrations of REEs were not significantly enhanced by the application of both DC and AC current (Fig. 4). However, the concentration of REEs was significantly increased in the ryegrass roots by applying AC current (Fig. 4) and, as a result, total plant concentrations, total plant uptake, and BCFs were also significantly improved (Table 2). This can be attributed to the increase in the secretion of root exudates induced by AC current, mainly organic acids that facilitate the chelation and availability of nutrients and REEs (and other metals) in the rhizosphere (Amari et al. 2017). As can be seen in Fig. 4, the highest mean shoot concentrations of REEs were obtained for the EKPhDC treatment while the highest REE concentrations in roots were reached under the EKPhAC one; it means that a higher root absorption did not necessarily lead to a higher shoot accumulation, indicating that REE translocation is hindered to some extent. Plants absorb REEs mainly in ionic form and their transport in soluble form to the aerial parts is carried out through the apoplastic (passive) and symplastic (active) pathways (Jalali and Lebeau 2021)*.* On the overall, the application of AC current increased the total plant concentrations of REEs between 63 and 75% and total plant uptake between 57 and 68%. AC current favored REE accumulation in the center of the soil containers, avoiding the transport to the electrode wells and thus facilitating contact between REEs and the ryegrass roots (Cameselle et al. 2013; Lima et al. 2017). Bi et al. identified an enhancement in the metal uptake mechanism in *Lactuca sativa* L. when a 50 Hz AC field was applied (Bi et al. 2010). This is related to the polarization and depolarization of the cell membrane in root cells caused by alternating current (Bi et al. 2011). The high frequency of this type of electric current causes changes in the voltage across the surface of the outer membrane and, consequently, the opening and closing of ionic channels and pumps, facilitating the diffusion of ions through the cell membrane; in turn, the negativity of the membrane surface potential is decreased, increasing the concentration of cations in the cell (Ehosioke et al. 2020).

Despite of the limited phyoextraction yields obtained in this study, it has been shown that phytomining could become a cost-effective technique to implement the recovery of valuable REEs from existing mine tailings, thus achieving the treatment of that type of hazardous waste and obtaining products of high-tech interest according to the principles of the circular economy (Van Loy et al. 2017). Moreover, the electrokinetic assistance clearly improved the phytoextraction potential of ryegrass that was the model plant species used in this study; the results obtained here make it worthwhile to explore the use of different conditions for the application of the electric field, mainly alternate current, as well as the use of potential REE hyperaccumulating plants in order to reach higher phytomining efficiencies (Siyar et al. 2020). Previous studies have suggested some species of hyperaccumulator ferns despite annual low dry matter production and the requirements of specific climatic conditions (Dinh et al. 2022). *Dicranopteris linearis* (Burm.f.) Underw (fern) is the most representative hyperaccumulator species due to its tolerance to the REE uptake from polluted soils; total concentrations of REEs (La, Ce, Pr, Nd, Sc, and Y) found in this plant accounted approximately 4400 and 1000 mg kg^−1^ for roots and shoots, respectively (Khan et al. 2017). The herbaceous perennial plant *Phytolacca americana* L. has also been detected to accumulate a total REE concentrations in the range 524–1040 mg kg^−1^ (Liu et al. 2020; Thomas et al. 2022). In the same way, some grasses, such as *Panicum miliaceum* L. and *Phalaris arundinacea* L., and herbs, such as *Fagopyrum esculentum* Moench and *Brassica napus* L., have demonstrated a convenient performance in REEs accumulation (Wiche and Heilmeier 2016). In any case, it is advisable to carry out an economic and environmental impact study to determine the cost–benefit ratio and consider phytomining with or without electrokinetic assistance as a feasible technique to recover REEs from mine waste.

## Conclusions

This work showed the results obtained in the application of electrokinetic-assisted phytoremediation to real mine tailings using both direct and alternating currents. It has been demonstrated the initial hypothesis that it is feasible to recover REEs from real metal mining waste by phytoextraction and that the performance of this technology can be significantly improved by applying electric current, especially of the AC type. Likewise, the mobilization of REEs in the mine tailings and the enhancement of their availability have been shown by combining the results obtained from the BCR sequential extraction, the analysis of the REE concentrations in the soil pore water and electrode well solutions, and their uptake by ryegrass. It was found that F1 and F2 BCR fractions are the most indicative of the availability of REEs for plant absorption and accumulation and how electric current along with plants can modify the distribution of those geochemical fractions, specially the F2 one. It is evident that the phytoextraction yields reported here are not sufficient to consider an economically feasible application of the studied technology to real cases. However, our results showed that it is interesting and recommendable to continue with the study of this technology for the recovery of REEs from mining waste and/or contaminated soils with special emphasis on the search for autochthonous hyperaccumulating plant species.

### Supplementary Information

Below is the link to the electronic supplementary material.

Supplementary file1 (DOCX 119 KB)

## Declarations

### Author consents of participation and publishing

All authors read and approved the final manuscript. All of them consent to both the participation and the publication of the work.
