# Supplementary material for: Can rare earth elements be recovered from abandoned mine tailings by means of electrokinetic-assisted phytoextraction?
Source: Environ Sci Pollut Res Int. 2024 Mar 8;31(18):26747–59. doi: 10.1007/s11356-024-32759-3 (PMC11052889; doi:10.1007/s11356-024-32759-3)
Supplement: Supplementary file 1 — Supplementary file1 (DOCX 119 KB) [file 11356_2024_32759_MOESM1_ESM.docx]

**Table SM1. Geochemical fractions obtained by modified sequential BCR extraction method (Rodríguez et al., 2009)**

| **Fractions** | **Soil phase** | **Reagent** |
| --- | --- | --- |
| **F1** | Exchangeable, water and acid-soluble (e.g., carbonates) | Acetic acid CH_3_COOH 99% |
| **F2** | Reducible (Bound to Fe/Mn oxides) | Hydroxyl ammonium chloride NH_2_OH⸱HCl 99% pH 1.5 |
| **F3** | Oxidizable (Bound to organic matter and sulfides) | Hydrogen peroxide H_2_O_2_ 30%  Ammonium acetate CH_3_COONH_4_ |
| **F4** | Residual (non-silicate bound metals) | EPA 3051A method: Nitric acid HNO_3_ 69%  Hydrochloric acid HCl 35% |

**Figure SM1. Effects of the different treatments on REEs concentrations (mg L^-1^) in the water obtained from electrode wells (W1 and W2) and the soil pores (collected from the middle section of each container by Rhizon samplers) sampled (n = 3) just before turning off the electric current on different days: (a) La; (b) Nd. Treatments applied: PHYTO: plants and no electricity; EKDC and EKAC: electric current and no plants; EKPhDC and EKPhAC: plants and electric current. For each REE and section; *, ** and *** indicate significant level (two-way ANOVA analysis) of ‘concentration x time’ interaction. Vertical bars show LSD test (p ≤ 0.05): (I) for same level of EK/Phyto treatment and (II) for different levels of treatments.**


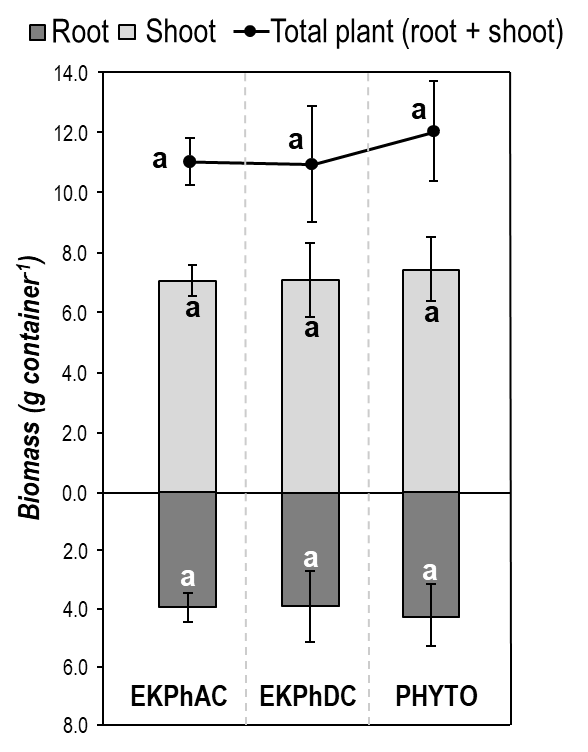


**Figure SM2. Mean values of ryegrass (*Lolium perenne*) dry matter (g per container) for roots, shoots and total biomass, at the end of the electrokinetic-assisted phytoextraction tests. Distinct letters indicate significant differences between treatments (EKPhAC, EKPhDC, and PHYTO) (n = 3; Fisher’s LSD test; p ≤ 0.05). Error bars represent the standard deviation of mean values.**
